# Supplementary material for: Reconciling Mining with the Conservation of Cave Biodiversity: A Quantitative Baseline to Help Establish Conservation Priorities
Source: PLoS One. 2016 Dec 20;11(12):e0168348. doi: 10.1371/journal.pone.0168348 (PMC5173368; doi:10.1371/journal.pone.0168348)
Supplement: S1 Dataset — (ZIP) [file pone.0168348.s002.zip › Taxa/Serra Norte/SN_2013/N4E/Lista N4E-74.pdf]

## CAVIDADE N4E-0074

| Classe | Ordem | Fam/Outros | Gên/Outros | Espécie | S-U | U-S | Zon. |
|---|---|---|---|---|---|---|---|
| Annelida | Oligochaeta | | | jovem | 5 | 3 | E/P |
| Arachnida | Acari | (Parasitengonina) | | sp.2 | | X | P |
| Arachnida | Acari | Cunaxidae | | sp.1 | X | | P |
| Arachnida | Acari | Prostigmata | | sp.49 | X | | P/A |
| Arachnida | Amblypygi | Charinidae | *Charinus* | sp.1 | 1 | 2 | P |
| Arachnida | Amblypygi | Phrynidae | *Heterophrynus* | *longicornis* | 3 | 4 | P/A |
| Arachnida | Araneae | Araneidae | *Alpaida* | *antonio* | | X | P |
| Arachnida | Araneae | Caponiidae | | jovem | | X | P |
| Arachnida | Araneae | Corinnidae | *Creugas* | sp.1 | 1 | 3 | E/P/A |
| Arachnida | Araneae | Ctenidae | | jovem | | 1 | |
| Arachnida | Araneae | Drymusidae | | jovem | | X | P |
| Arachnida | Araneae | Ochyroceratidae | *Ochyrocera* | sp.3 | | X | A |
| Arachnida | Araneae | Ochyroceratidae | *Speocera* | sp.1 | | X | P |
| Arachnida | Araneae | Ochyroceratidae | | jovem | X | X | P |
| Arachnida | Araneae | Oonopidae | aff. *Xyccarph* | sp.2 | X | | P |
| Arachnida | Araneae | Oonopidae | *Scaphiella* | sp.1 | | X | P |
| Arachnida | Araneae | Pholcidae | *Leptopholcus* | sp.1 | X | | E |
| Arachnida | Araneae | Pholcidae | *Mesabolivar* | sp.1 | X | X | P |
| Arachnida | Araneae | Salticidae | | jovem | | X | P |
| Arachnida | Araneae | Theridiosomatidae | *Plato* | sp.1 | X | X | P/A |
| Arachnida | Opiliones | Cosmetidae | *Roquettea* | *singularis* | X | X | |
| Arachnida | Opiliones | Escadabiidae | | sp.2 | | X | P |
| Arachnida | Pseudoscorpiones | Bochicidae | | sp.1 | | X | A |
| Arachnida | Pseudoscorpiones | Chthoniidae | *Pseudochthonius* | sp.1 | | X | P |
| Arachnida | Schizomida | Hubbardiidae | aff. *Rowlandius* | sp.1 | X | X | P |
| Chilopoda | Scolopendromorpha | Cryptopidae | *Cryptops* | jovem | | 1 | P |
| Chilopoda | Scolopendromorpha | Scolopocryptopidae | *Dinocryptops* | *miersii* | | 1 | A |
| Diplopoda | Polydesmida | Chelodesmidae | | jovem | 1 | | E |
| Diplopoda | Polydesmida | Cyrtodesmidae | | sp.1 | X | | E |
| Diplopoda | Polydesmida | Pyrgodesmidae | | sp.1 | X | X | P/A |
| Diplopoda | Siphonophorida | Siphonophoridae | | sp.1 | X | | E |
| Entognatha | Collembola | Cyphoderidae | | sp.1 | X | | E |
| Entognatha | Collembola | Entomobryidae | | sp.4 | | X | P |
| Entognatha | Collembola | Paronellidae | | sp.1 | X | | E |
| Entognatha | Collembola | Paronellidae | | sp.4 | | X | P |
| Entognatha | Collembola | Sminthuroidea | | sp.2 | X | X | P/A |
| Entognatha | Diplura | Campodeidae | | sp.1 | X | X | E/P |
| Entognatha | Diplura | Japygidae | | sp.2 | | X | P |
| Entognatha | Diplura | Projapygidae | | sp.1 | X | X | P |
| Insecta | Blattodea | Blattellidae | | jovem | 3 | 7 | A |
| Insecta | Coleoptera | Carabidae | | sp.3 | | X | A |
| Insecta | Coleoptera | Scydmaenidae | | sp.1 | | X | P |
| Insecta | Coleoptera | Scydmaenidae | | sp.6 | | X | P |
| Insecta | Coleoptera | Staphylinidae | Pselaphinae | sp.25 | X | | E |
| Insecta | Dermaptera | Anisolabididae | | sp.1 | 1 | | E |
| Insecta | Diptera | Chamaemidae | | sp. | X | | E/A |
| Insecta | Diptera | Culicidae | *Culex* | sp. | X | X | P/A |
| Insecta | Diptera | Dolichopodidae | | sp. | | X | P |
| Insecta | Diptera | Psychodidae | *Breviscapus* | sp. | X | | A |

| | | | | | | | |
|---|---|---|---|---|---|---|---|
| Insecta | Diptera | Psychodidae | Phlebotominae | sp. | X | X | P/A |
| Insecta | Diptera | Tipulidae | Tipulinae | sp. | | X | P |
| Insecta | Heteroptera | Cydnidae | | jovem | X | | A |
| Insecta | Heteroptera | Reduviidae | *Panstrongylus* | sp.1 | 3 | | |
| Insecta | Heteroptera | Reduviidae | Reduviinae | sp. | | 2 | P/A |
| Insecta | Homoptera | Cixiidae | | sp.3 | | X | P |
| Insecta | Hymenoptera | Formicidae | *Paratrechina* | sp.1 | X | | P |
| Insecta | Hymenoptera | Formicidae | *Camponotus* | sp.2 | X | X | P |
| Insecta | Hymenoptera | Formicidae | *Gnamptogenys* | sp.1 | X | X | P |
| Insecta | Hymenoptera | Formicidae | *Hypoponera* | sp.2 | | X | P |
| Insecta | Hymenoptera | Formicidae | *Pachycondyla* | sp.2 | X | X | E/P/A |
| Insecta | Hymenoptera | Formicidae | *Paratrechina* | sp.2 | X | | P/A |
| Insecta | Hymenoptera | Formicidae | *Solenopsis* | sp.1 | | X | P |
| Insecta | Isoptera | Termitidae | *Nasutitermes* | sp. | X | X | E/P/A |
| Insecta | Lepidoptera | Noctuoidea | | sp.5 | | 1 | A |
| Insecta | Lepidoptera | Noctuoidea | | sp.7 | | 2 | P |
| Insecta | Lepidoptera | Roeslerstammiidae | | sp. | | X | P |
| Insecta | Orthoptera | Phalangopsidae | *Paraclodes* | jovem | | 11 | E |
| Insecta | Orthoptera | Phalangopsidae | *Phalangopsis* | sp. | 192 | 305 | P/A |
| Insecta | Psocoptera | | | jovem | X | | E/P |
| Insecta | Thysanoptera | Thripidae | | sp.1 | | X | A |
| Insecta | Thysanura | Nicoletiidae | | sp.1 | X | X | P/A |
| Gastropoda | Pulmonata | Bulimulidae | *Naesiotus* | sp. | X | X | P/A |
| Malacostraca | Isopoda | Philosciidae | | sp.2 | X | | E |
| Malacostraca | Isopoda | Platyarthridae | | sp.1 | | X | P |
| Malacostraca | Isopoda | Scleropactidae | *Circoniscus* | *buckupi* | X | | P |
| Symphyla | | Scutigerellidae | | sp. | X | X | E/P/A |
| Turbellaria | Tricladida | | | sp.2 | | 4 | P |
| Amphibia | Anura | Strabomantidae | *Pristimantis* | cf. *fenestratus* | 7 | | P |
| Mammalia | Chiroptera | Emballonuridae | *Peropteryx* | *kappleri* | 2 | 3 | P |
| Mammalia | Chiroptera | Furipteridae | *Furipterus* | *horrens* | 4 | 20 | A |
| Mammalia | Chiroptera | Phyllostomidae | *Carollia* | *perspicillata* | | 10 | A |
| Mammalia | Chiroptera | Phyllostomidae | *Diphylla* | *ecaudata* | | 15 | P |
